# Multi-omics analysis reveals associations between gut microbiota and host transcriptome in colon cancer patients

**DOI:** 10.1128/msystems.00805-24

**Published:** 2025-02-27

**Authors:** Yuling Qin, Qiang Wang, Qiumei Lin, Fengfei Liu, Xiaolan Pan, Caibiao Wei, Junxian Chen, Taijun Huang, Min Fang, Weilong Yang, Linghui Pan

**Affiliations:** 1 Department of Clinical Laboratory, Guangxi Medical University Cancer Hospital 117981 , Nanning, China; 2 Guangxi Clinical Research Center for Anesthesiology, Guangxi Medical University Cancer Hospital 117981 , Nanning, China; 3 Guangzhou Women and Children’s Medical Center, Guangzhou Medical University 26468 , Guangzhou, China; 4 Institute of Advanced Biotechnology and School of Medicine, Southern University of Science and Technology 255310 , Shenzhen, China; University of California, San Francisco, San Francisco, California, USA

**Keywords:** multi-omics, colon cancer, mucosal microbiota, correlation, bile secretion, immune, survival value

## Abstract

Colon cancer (CC) is one of the most common cancers globally, which is associated with the gut microbiota intimately. In current research, exploring the complex interaction between microbiomes and CC is a hotspot. However, the information on microbiomes in most previous studies is based on fecal, which does not fully display the microbial environment of CC. Herein, we collected mucosal and tissue samples from both the tumor and normal regions of 19 CC patients and clarified the composition of mucosal microbiota by 16S rRNA and metagenomic sequencing. Additionally, RNA-Seq was also conducted to identify the different expression genes between tumor and normal tissue samples. We revealed significantly different microbial community structures and expression profiles to CC. Depending on correlation analysis, we demonstrated that 1,472 genes were significantly correlated with CC tumor microbiota. Our study reveals a significant enrichment of *Campylobacter jejuni* in the mucosa of CC, which correlates with bile secretion. Additionally, we observe a negative correlation between *C. jejuni* and immune cells CD4+ Tem and mast cells. Finally, we discovered that metabolic bacterial endosymbiont of *Bathymodiolus* sp., *Bacillus wiedmannii*, and *Mycobacterium tuberculosis* had a significant survival value for CC, which was ignored by previous research. Overall, our study expands the understanding of the complex interplay between microbiota and CC and provides new targets for the treatment of CC.

**IMPORTANCE:**

This study contributes to our understanding of the interaction between microbiota and colon cancer (CC). By examining mucosal and tissue samples rather than solely relying on fecal samples, we have uncovered previously unknown aspects of CC-associated microbiota. Our findings reveal distinct microbial community structures and gene expression profiles correlated with CC progression. Notably, the enrichment of *Campylobacter jejuni* in CC mucosa, linked to bile secretion, underscores potential mechanisms in CC pathogenesis. Additionally, observed correlations between microbial taxa and immune cell populations offer new avenues for immunotherapy research in CC. Importantly, this study introduces CC-associated microbiota with survival implications for CC, expanding therapeutic targets beyond conventional strategies. By elucidating these correlations, our study not only contributes to uncovering the potential role of gut microbiota in colon cancer but also establishes a foundation for mechanistic studies of gut microbiota in colon cancer, emphasizing the broader impact of microbiota research on cancer biology.

## INTRODUCTION

Colon cancer (CC) is the third most common cancer worldwide, causing nearly 1 million deaths annually (1). While advancements in diagnosing and treating CC have been achieved, leading to a reduction in mortality rates, the 5 years survival rate remains notably low (2). Environmental factors and hereditary factors both contribute to colon cancer. However, such as Lynch syndrome and familial adenomatous polyposis, the genetic susceptibility factors only account for a minority of CC cases (3, 4). Therefore, the impact of environmental factors on colon cancer is significant.

The role of microorganisms in the realm of cancer biology, within the influence of environmental factors, is progressively gaining recognition (5). Gut microbiota associates with colon cancer intimately, forming a unique tumor microbiota, suggesting the significant role of tumor-associated microbiota in CC (6). In general, CC patients frequently exhibit the characteristic of dysbiosis in the gut microbiome (7). The findings of the current study indicate a strong correlation between the occurrence and development of CC and certain bacterial species, such as *Fusobacterium*, *Porphyromonas*, and *Bifidobacterium*, among others (8–10). For instance, in colorectal cancer (CRC), *Fusobacterium nucleatum* promotes glycolysis and tumorigenesis by targeting lncRNA ENO1-IT1 (11). *Porphyrinomonas* promotes tumorigenesis utilizing the bacterial metabolite butyrate secretion (12). The activation of the hematopoietic NLRP3 inflammasome by *Porphyromonas gingivalis* is implicated in the promotion of colorectal cancer (13). Enterotoxigenic *Bifidobacterium fragilis* toxins promote tumor occurrence by activating STAT3 and Wnt pathways and stimulating IL-17 production (14).

In previous studies, the information on gut microbiota in CC was mostly based on fecal matter (15, 16). However, fecal samples can only partially reflect the composition of the intestinal microbiota and there are significant differences between fecal microbiota and intestinal microbiota (17, 18). The research by Li et al. suggests that certain genera, including *Fusobacterium* and *Gemella*, exhibit a greater propensity for adherence to tumor mucosa as opposed to being excreted in significant quantities within fecal matter (19). The evolution of intestinal mucosal microbiota is closely linked to the host and forms a specific local community. Intestinal mucosal microbiota is important for host health and disease development and can accurately describe the microbiota associated with colorectal cancer (20). Additionally, a complex interplay exists between the gut microbiota and host gene expression (21–23). The synergistic investigation of host transcriptomics and microbiome explains host-microbiota interaction at the gene expression level and shows gut microbiota-differential gene interaction mechanism. Therefore, in this study, we analyzed the different microbial community structures of CC based on mucosa using 16S rRNA and metagenomic sequencing. Subsequently, we identified differentially expressed genes (DEGs) and demonstrated their correlation with tumor-associated microbiota. Finally, we evaluated the survival value of tumor-associated microbiota in colon cancer. Overall, this comprehensive multi-omics approach helps us comprehend the complicated link between gut microbiota, host gene expression, and colon cancer, ultimately leading to potential insights for future therapeutic interventions.

## MATERIALS AND METHODS

### Study subjects and sample collection

The human specimens involved in this study were obtained from clinical tissue samples of patients who had radical resection of colon cancer tumors at the Cancer Hospital Affiliated to Guangxi Medical University from January to May 2022. The criteria for patient inclusion were as follows: (i) confirmed by colonoscopy pathology, the patient is diagnosed with colon cancer for the first time; (ii) the patient should be at least 18 years old. The criteria for exclusion are as follows: (i) recurrent colon cancer patients; (ii) having undergone any form of chemotherapy and radiation therapy before surgery; (iii) cancer concurrent with other systems. All patients included in this study have not undergone any antibiotic therapy within the past month. Paired normal and tumor tissues and mucosa were collected during open surgery, and normal tissues or mucosa were obtained at a distance of at least 10 cm from the tumor tissue. Table S1 details 38 samples from 19 patients in this study.

Postoperative fresh surgical specimens were washed with sterile saline solution to gently remove fecal material adhering to the mucosal surface. Sterile scalpels and cotton swabs were used to scrape the mucosal surface on the tumor site and at least 10 cm away from the tumor site. The samples were stored in −80°C frozen tubes for 16S rRNA and metagenomic sequencing. Tissue samples from the same site were collected using sterile tissue scissors and stored in −80°C frozen tubes for transcriptomic sequencing. After collection, the specimens were transported on dry ice to Novogene Co., Ltd., Beijing, for processing and sequencing.

### 16S rRNA sequencing and analysis

The bacterial DNA was isolated from samples of colon contents using a Magnetic Soil and Stool DNA Kit (Tiangen Biotech, China) following the instructions provided by the manufacturer. The concentration and purity of DNA were assessed using a 1% agarose gel. 16S rRNA/18S rRNA/ITS genes in distinct regions (16S V4/16S V3/16S V3-V4/16S V4-V5, 18S V4/18S V9, ITS1/ITS2, Arc V4) were amplified with specific primers (e.g., 16S V4: 515F-806R, 18S V4: 528F-706R, 18S V9: 1380F-1510R, etc.) and barcodes. The PCR-amplified products were detected and purified by 2% agarose gel electrophoresis with a Qiagen Gel Extraction Kit (Qiagen, Germany). Sequencing libraries were constructed using TruSeq DNA PCR-Free Sample Preparation Kit (Illumina, USA) according to the instructions provided by the manufacturer. The library underwent sequencing using an Illumina NovaSeq platform, resulting in the generation of 250 bp paired-end reads. The primer sequences used for sequencing were as follows: GTGCCAGCMGCCGCGGTAA, GGACTACHVGGGTWTCTAAT (24, 25).

High-quality clean data were obtained by subjecting the raw data to quality filtering using particular filtering conditions, following the QIIME (26, 27) (V1.9.1, http://qiime.org/scripts/split_libraries_fastq.html) quality-controlled process. Based on the clean data, the process of clustering operational taxonomic units (OTUs) and classifying species was evaluated using Uparse (28) (V7.0.1001, http://www.drive5.com/uparse/). The Silva Database (29) (http://www.arb-silva.de/) was utilized, employing the Mothur algorithm, to annotate taxonomic information for each representative sequence. The abundance information of OTUs was normalized using a standard based on the sequence number corresponding to the sample with the least sequences.

Alpha diversity indices including observed species, Chao1, Shannon, Simpson, ACE, and Good’s coverage were calculated with QIIME (V1.7.0) and displayed with R software (V2.15.3). Beta diversity among different groups was detected based on the unweighted UniFrac index using QIIME (V1.9.1) software.

### Metagenomic sequencing and analysis

The extraction of bacterial DNA from samples of colon contents was performed using a Magnetic Soil and Stool DNA Kit (Tiangen Biotech, China) in accordance with the manufacturer’s instructions. The genomic DNA underwent random shearing, resulting in the formation of short fragments. The obtained fragments underwent end-repair, A-tailing, and subsequent ligation with an Illumina adapter. The adapter-containing fragments were PCR amplified, size chosen, and purified. Qubit and real-time PCR were used to quantify, and a bioanalyzer was used to detect size distribution in the library. The quantified libraries were pooled and subjected to sequencing using Illumina PE150 platforms, based on the optimal library concentration and data amount required.

Raw data from the Illumina sequencing platform were preprocessed with Readfq (V8, https://github.com/cjfields/readfq) to create clean data for analysis. The assembly analysis of clean data was conducted using the MEGAHIT software (V1.0.4-beta). Starting from the assembled scaftigs of individual samples, gene prediction was performed using MetaGeneMark (V3.05, http://topaz.gatech.edu/GeneMark/). The predicted genes from each sample were then compiled, and redundancies were removed to construct a gene catalog (Unigenes). DIAMOND (30) software (V0.9.9, https://github.com/bbuchfink/diamond/) was used to align Unigenes with those in the functional database. Functional databases include Kyoto Encyclopedia of Genes and Genomes (31, 32) (KEGG, V2018-01-01, http://www.kegg.jp/kegg/), Evolutionary Genealogy of Genes: Non-supervised Orthologous Groups (33) (eggNOG, V4.5, (http://eggnogdb.embl.de/#/app/home), and Carbohydrate-active Enzymes Database (34) (CAZy, V201801, http://www.cazy.org/). Based on Bray-Curtis distance, non-metric multi-dimensional scaling (NMDS) analysis (35) was conducted using the vegan, permute, lattice, ade4, ggplot2, and grid packages within R (V1.9.1). Analysis of similarities (ANOSIM) was used to test the differences between groups (R vegan package, V2.15.3). The rarefaction curves, species accumulation boxplot, and principal coordinates analysis (PCoA) were generated using the FactoMineR, WGCNA, stats, and ggplot2 packages in R software (V3.2.1). The Linear discriminant analysis Effect Size software (LEfSe, V1.0) (36) was utilized to perform LEfSe analysis, with a Linear Discriminant Analysis (LDA) score threshold of 3, in order to identify the biomarkers.

### RNA sequencing and analysis

Colonic contents were subjected to RNA extraction using the Trizol method (Ambion, USA). The quantification and quality assessment of RNA samples was performed using the RNA Nano 6000 Assay Kit on the Bioanalyzer 2100 instrument (Agilent Technologies, CA, USA). The RNA sample preparations utilized total RNA as the input material. To isolate cDNA fragments with a desired length range of 370–420 bp, the library fragments underwent purification using the AMPure XP system (Beckman Coulter, Beverly, USA). Following the PCR amplification, the resulting PCR product underwent purification using AMPure XP beads, resulting in the library. After qualification, the libraries are pooled by effective concentration and the target amount of data off the machine, then sequenced by Illumina NovaSeq 6000. The end reading of 150 bp pairing is generated.

The sequencing data underwent quality control and filtration processes. Hisat2 (37) (V2.0.5) was used to create the reference genome index and match paired-end clean reads to it. FeatureCounts (38) (V1.5.0-p3) was employed for quantifying the number of mapped reads for each gene. Subsequently, each gene’s FPKM (Fragments Per Kilobase of transcript per Million mapped fragments, FPKM) value was calculated from its length and read count. To analyze differential expression between two groups, the DESeq2 (39) R package was utilized. Padj ≤ 0.05 and |log2(foldchange)| ≥ 1 were set as the threshold for significantly differential expression. Clusterprofiler (40) (V3.8.1) software was used to analyze the statistical enrichment of differentially expressed genes in the KEGG pathway. Based on the algorithm provided in R package xCell (41) (V4.2.1), the immune infiltration of each sample is calculated by using the markers of 64 kinds of immune cells provided internally.

### Correlation analysis

Based on the differentially expressed genes, immune infiltration score, and the absolute abundance of differential microbiota, the psych package in R (V4.4.0) software was used for Spearman correlation analysis and significance test. According to the correlation coefficient, corrplot package was used to draw the correlation heat map. The conditions for screening-related genes were *P* < 0.05, *r* ≥ 0.5, or *r* ≤ −0.5.

### Survival analysis

Firstly, we used GEPIA2 (42) (http://gepia2.cancer-pku.cn/#index) to build the survivorship curve of DEGs in the passageways of bile secretion. To do a batch survival analysis of all DEGs, the phenotypic information and gene expression matrix of GDC TCGA Colon Cancer (COAD) were downloaded from the TCGA database. We took the intersection of the DEGs and the gene expression matrix from the TCGA database, and found that only 1,365 genes overlapped. Survival R package (43) was used to do survival analysis, and *P* < 0.05 was set as the threshold for a significant survival rate.

## RESULTS

### Disordered mucosal microbiota structures of CC

**Fig 1:**
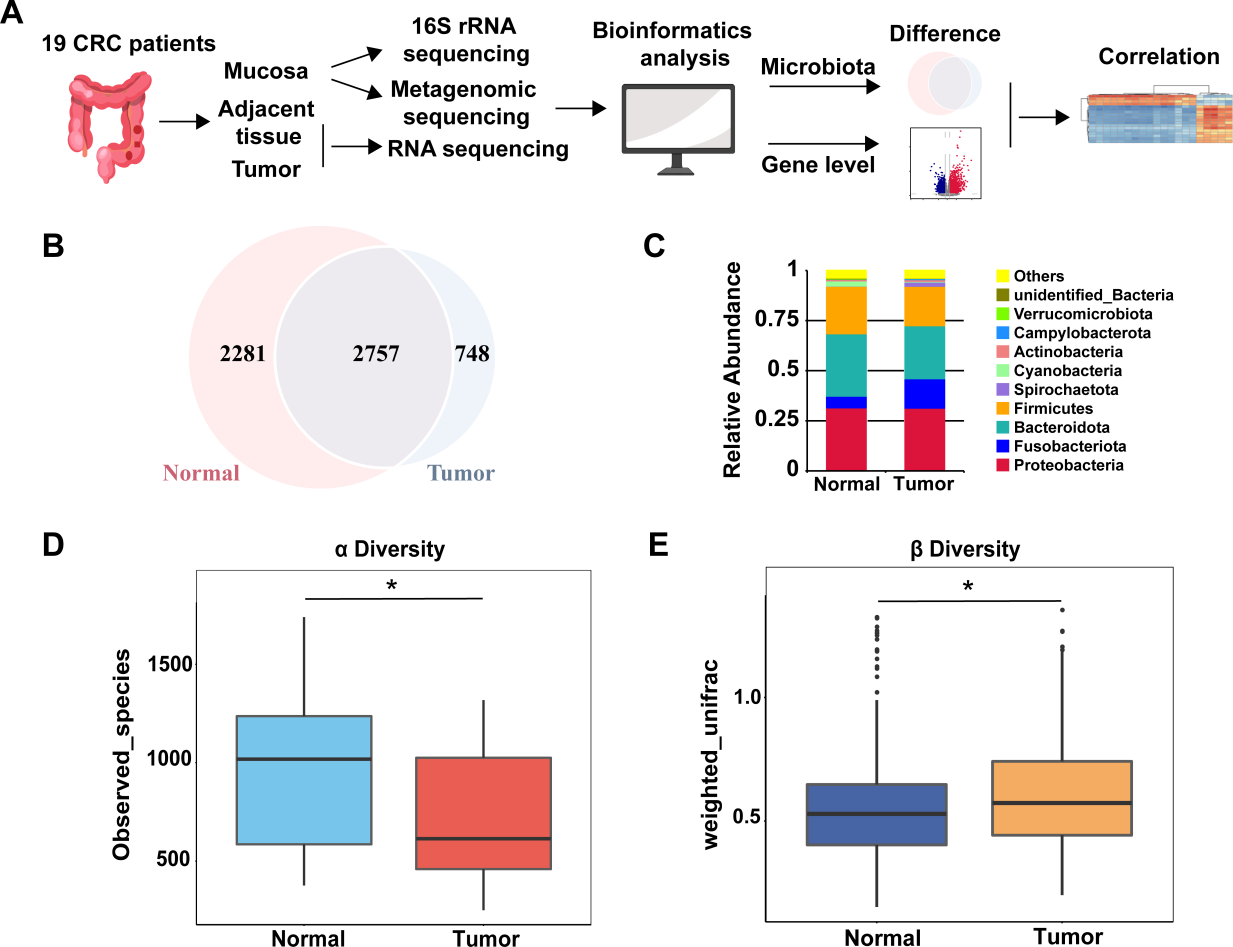
Disordered mucosal microbiota of CC based on 16S rRNA sequencing. (**A**) The schematic of the overall approach. (**B**) Venn diagram for the number of unique and common OTUs in normal and tumor groups. (**C**) The mucosal microbiota composition of normal and tumor groups at the phylum level. (**D**) Alpha diversity index between groups difference analysis. (**E**) Beta diversity index between groups difference analysis. *P < 0.05.

To investigate the influence of microbial microenvironment on CC, a workflow was designed (Fig. 1A). Firstly, 21 pairs of tumor and normal mucosal samples were obtained to do 16S rRNA sequencing (Table S2). After quality-control filtering, 19 pairs of sequence data were remained. The species accumulation boxplot and rarefaction curve show that the sample size of our study is sufficient, and the amount of sequencing data is reasonable (Fig. S1). Clustering yielded 5,786 OTUs, 2,281 of which were unique to normal and 748 to tumor groups (Fig. 1B). According to the taxonomic annotations, at the phylum level, the two microbial communities are mainly composed of the *Proteobacteria* (N: 31.58%; T: 31.45%), *Bacteroidota* (N: 31.15%; T: 26.44%), *Firmicutes* (N: 23.70%; T: 19.69%), and *Fusobacteriota* (N: 5.83%; T: 14.68%) (Fig. 1C; Fig. S2).

Based on the analysis of alpha diversity using the observed species index, there was a significant difference in species richness between the normal group and the tumor group (Fig. 1D, *P* < 0.05; Table S3). In addition, this study compared the diversity of gut bacteria using the β-diversity index and found significant differences in the composition of gut microbiota between the two groups (Fig. 1E, *P* < 0.05).

### Annotation of tumor-associated microbiota and functions

**Fig 2:**
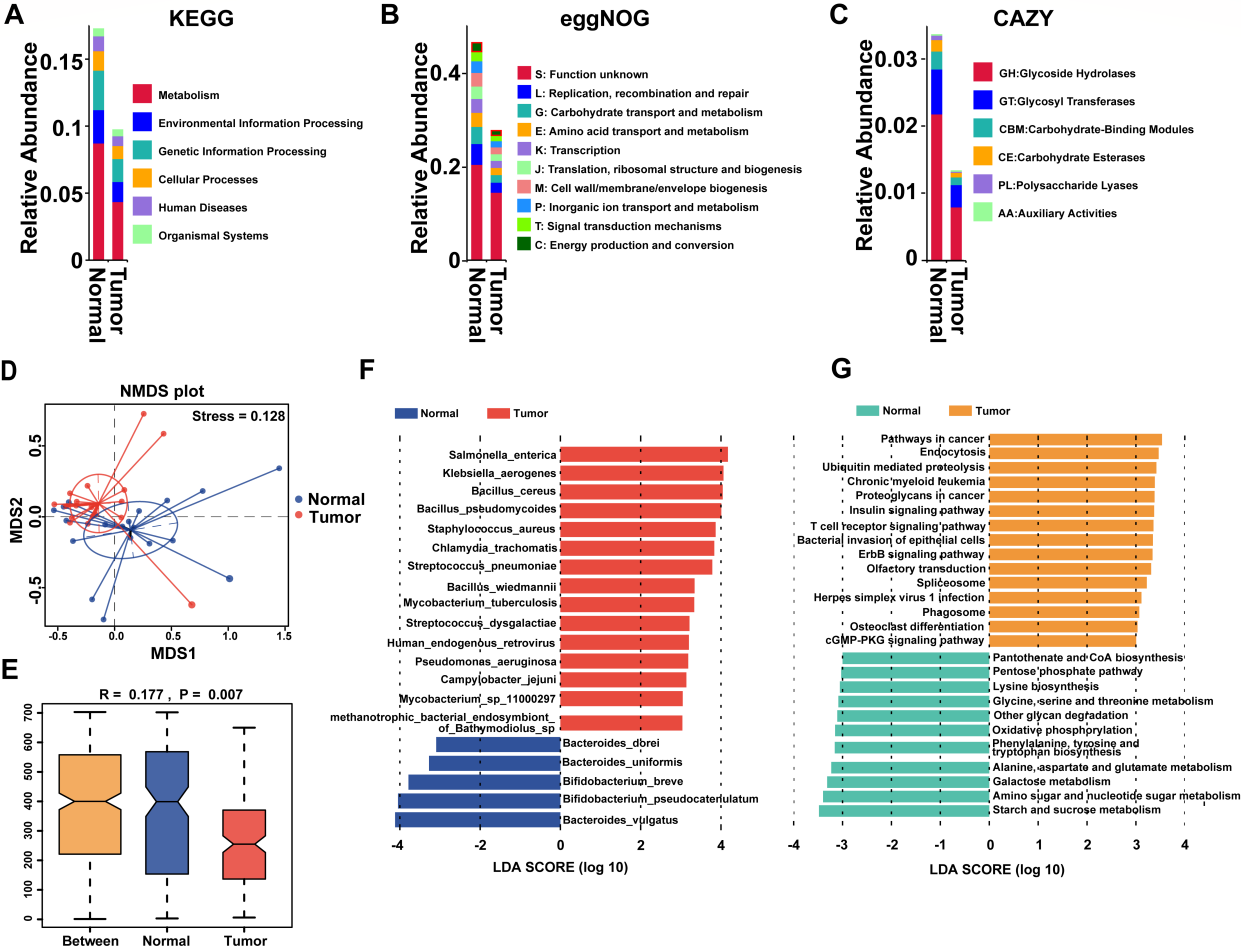
Function analysis of mucosal microbiota based on metagenomic sequencing. (**A–C**) At the first classification level, the functional composition of mucosal microbiota in normal and tumor groups at the KEGG (**A**), eggNOG (**B**), and CAZy (**C**) databases. (**D**) NMDS analysis of normal and tumor groups based on the species of phylum level. (**E**) ANOSIM of normal and tumor groups based on the species of phylum level. (**F**) Distribution histogram of LDA values of different species (LDA > 3). (**G**) Distribution histogram of LDA based on functional abundance (LDA > 3).

To further elucidate the functional composition of colon cancer mucosal microbiota, we conducted metagenomic sequencing and functional annotation on 19 pairs of tumor and normal mucosal samples (Table S4). The core-pan gene dilution curve and the heatmap of gene abundance correlation among samples demonstrated the reliability of the experiment and the rationality of sample selection (Fig. S3). All functional annotation results, based on KEGG, eggNOG, and CAZy databases, showed that the functional abundance composition was different between the normal and tumor groups (Fig. 2A through C; Fig. S4). PCoA and ANOSIM illustrated the obvious separation of microbial functional composition between normal and tumor groups (Fig. S5), confirming that there were significant functional abundance differences between normal and tumor groups. Based on the CAZy database, we also explored the abundance differences of different enzymes (Tables S5 and S6). NMDS and ANOSIM based on species abundance revealed that there were significant differences in species composition between normal and tumor groups at the phylum level (Fig. 2D and E).

Next, in order to screen for species and functional biomarkers with significant differences between groups, we conducted LEfSe analysis. It is worth noting that we identified 20 differentially abundant species associated with the disease state, and we defined them as tumor-associated microbiota (Fig. 2F). The LEfSe analysis based on species abundance shows that species such as *Bacteroides vulgatus*, *Bifidobacterium pseudocatenulatum*, *Bifidobacterium breve*, *Bacteroides uniformis*, and *Bacteroides dorei* are enriched in the normal group. Species such as *Salmonella enterica*, *Klebsiella aerogenes*, *Bacillus cereus*, *Bacillus pseudomycoides*, and *Staphylococcus aureus* are enriched in the tumor group. Based on the KEGG databases, we found that starch and sucrose metabolism (ko00500), amino sugar and nucleotide sugar metabolism (ko00520), and galactose metabolism (ko00052) were significantly more abundant in the normal group than in the tumor group (Fig. 2G). However, the top three upregulated pathways in the tumor group were pathways in cancer (ko05200), endocytosis (ko04144), and ubiquitin-mediated proteolysis (ko04120).

### Altered gene expression profile in CC

**Fig 3:**
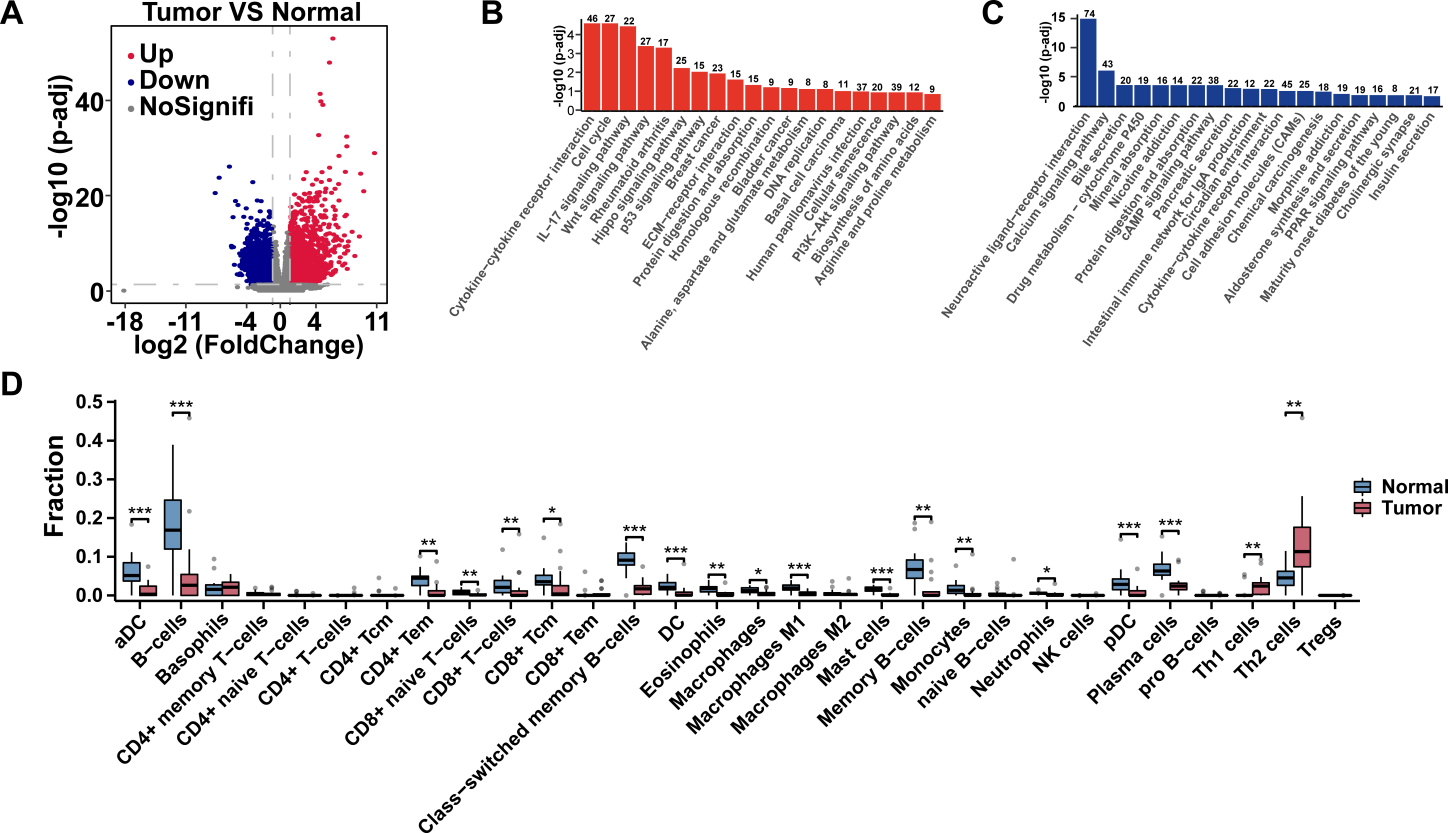
Different transcriptome profiles between normal and tumor groups. (**A**) The DGEs between normal and tumor groups. (**B**) The KEGG enrichment analysis of up-expression genes in tumor groups. (**C**) The KEGG enrichment analysis of down-expression genes in tumor groups. (**D**) Analysis of immune infiltration between the normal group and the tumor group. *p < 0.05, **p < 0.01, ***p < 0.001.

In order to uncover the different gene expressions of CC, 19 pairs of tumor and adjacent non-cancerous tissues (normal) were obtained to do RNA-Seq (Table S7). After stringent data quality control and filtering, one pair of samples was excluded. Transcriptome analysis showed that there were 4,693 DEGs identified in the tumor group, compared to the normal group (|log2(foldchange)| ≥ 1 and Padj ≤ 0.05) (Fig. 3A), including 2,169 up-expression genes and 2,524 down-expression genes. Next, we also do KEGG pathway clustering analysis to up-expression genes and down-expression genes. We found that up-expression genes were mainly enriched in cytokine-cytokine receptor interaction, cell cycle, IL-17 signaling pathway, etc., and down-expression genes were mainly enriched in neuroactive ligand-receptor interaction, calcium signaling pathway, bile secretion, etc. (Fig. 3B and C). In addition, differential analysis based on immune infiltration showed differences in 30 types of immune cells between the normal group and the tumor group (Fig. 3D).

### Correlation between tumor-associated microbiota and DEGs in CC

**Fig 4:**
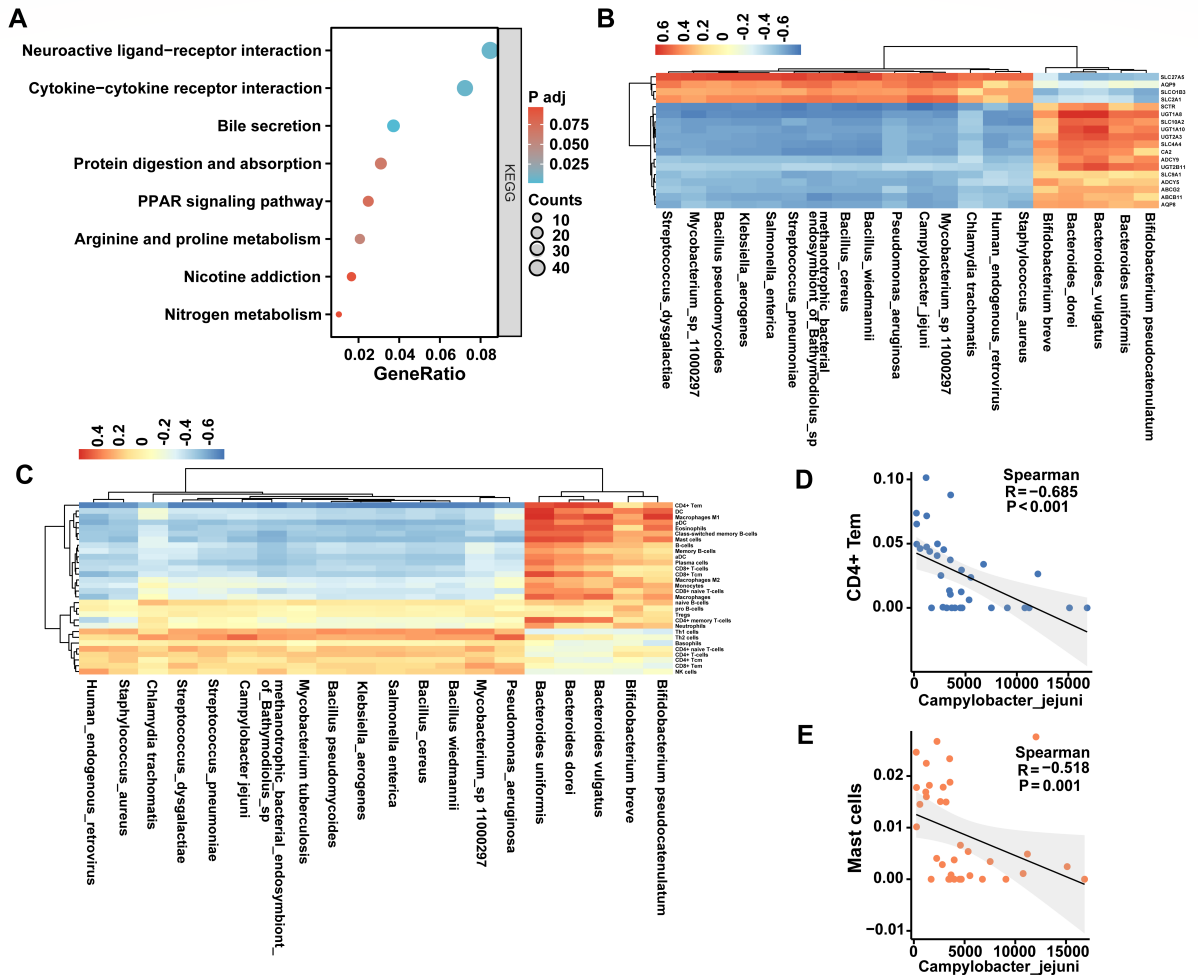
Correlation between tumor-associated microbiota and DEGs. (**A**) The KEGG enrichment analysis of tumor-associated microbiota related DEGs. (**B**) Correlation between tumor-associated microbiota and 18 bile secretion related DEGs. (**C**) Correlation between tumor-associated microbiota and 30 immune cells. (**D and E**) The correlation between *Campylobacter jejuni* and CD4+ Tem (**D**) and master cells (E).

The tumor-associated microbiota is strongly related to CC; however, its relationship to host gene expression is still unclear (44). Therefore, we determined the correlation between the tumor-associated microbiota and the 3,632 DEGs (Padj < 0.01) identified previously. Among them, 1,472 DEGs were significantly correlated to the tumor-associated microbiota (20 species, LDA > 3) (Table S8). Notably, the number of correlated DEGs in each tumor-associated microbiota genus was different, ranging from 86 to 872 (Fig. S6). KEGG pathway clustering analysis was performed on these 1,472 correlated DEGs, indicating that they were significantly enriched in eight functional pathways (bile secretion, neuroactive ligand-receptor interaction, cytokine-cytokine receptor interaction, arginine and proline metabolism, protein digestion and absorption, PPAR signaling pathway, nicotine addiction, Nitrogen metabolism) (Fig. 4A).

Previous studies showed that bile secretion had a relationship with CC (45). Thus, we picked up all 18 DEGs related to bile secretion and highlighted their correlation to 20 tumor-associated microbiota (Fig. 4B). It demonstrated that *Campylobacter jejuni* and methanotrophic bacterial endosymbiont of *Bathymodiolus* sp. had strong correlations to bile secretion (Fig. S7). In addition, based on species abundance and transcriptome immune infiltration results, we also explored the correlation between different species and host immunity (Fig. 4C). It is worth mentioning that there is a negative correlation between *Campylobacter jejuni* and CD4+ Tem and master cells (Fig. 4D and E).

### Tumor-associated microbiota has a significant survival value for CC

**Fig 5:**
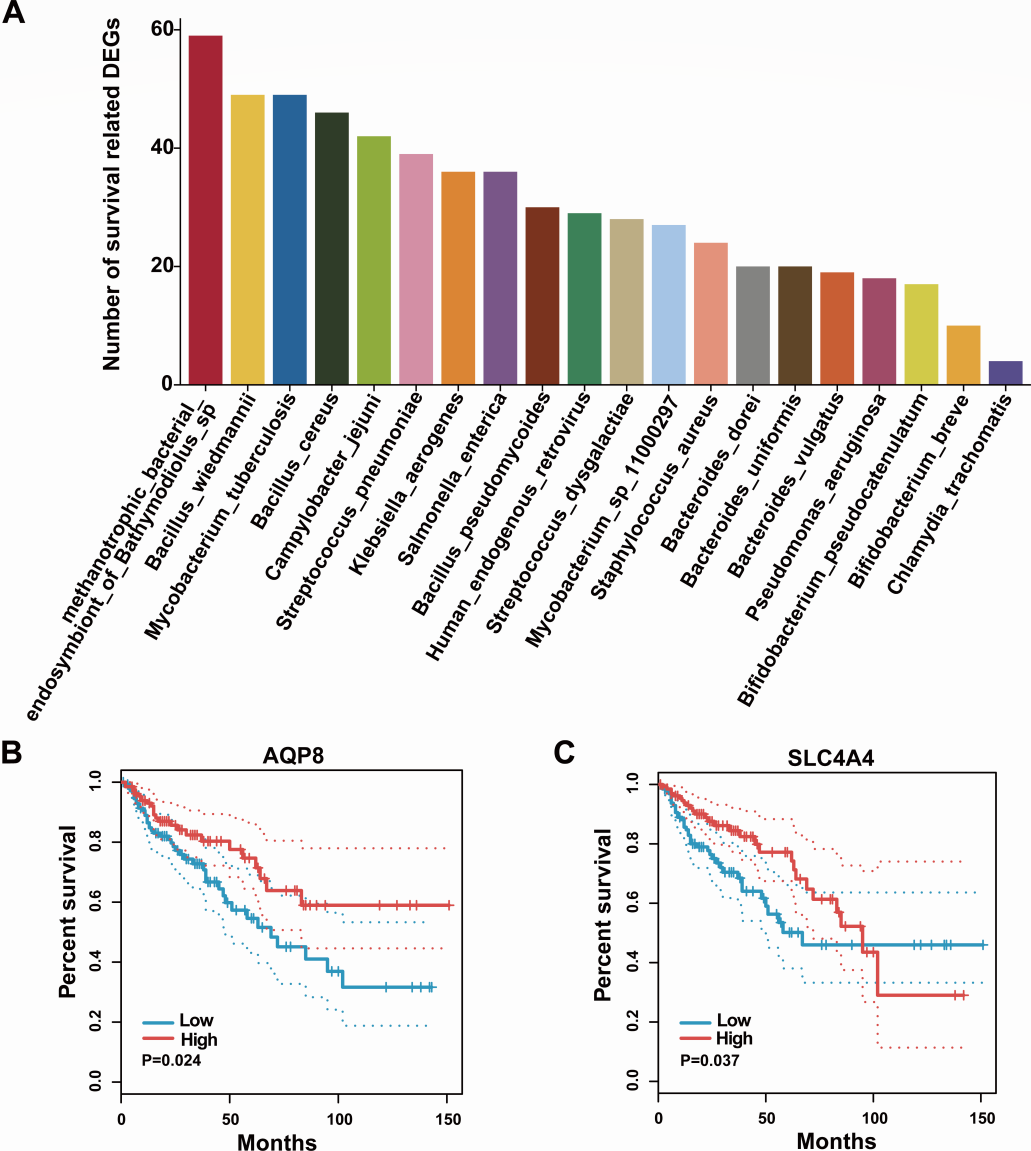
Survival analysis of DEGs and tumor-associated microbiota. (**A**) The number of DGEs which were both associated with survival and correlated to the tumor-associated microbiota at the species level. (**B and C**) Survival analysis of AQP8 (**B**) and SLC4A4 (C).

Although 1,472 DEGs were found significantly correlated to the tumor-associated microbiota above, most of these DEGs did not influent the survival of CC patients. Therefore, we did the survival analysis of all DEGs in CC based on the TCGA database, and 100 DEGs were identified as related to the survival of CC patients (*P* < 0.05) (Table S9). Next, we calculated the number of correlated DEGs related to the survival of CC patients in each tumor-associated microbiota. The number of each species had an obvious difference (Fig. 5A; Table S10). Among them, compared to the other tumor-associated microbiota species, methanotrophic bacterial endosymbiont of *Bathymodiolus* sp., *Bacillus wiedmannii*, and *Mycobacterium tuberculosis* have more DEGs related to the survival of CC patients. This means that they have significant survival value for CC. Importantly, we also performed the survival analysis of all 18 DEGs related to bile secretion in CC, revealing that AQP8 and SLC4A4 were related to the survival of CC patients (Fig. 5B and C).

## DISCUSSION

### Portray tumor-associated microbiota accurately

The tumor-associated microbial community is closely linked to the occurrence and development of tumors, and the presence of these microorganisms has an impact on tumor progression and treatment response (46, 47). Fecal microbes are used to do most of the related research; however, many studies have shown that there are significant differences in the bacterial composition between fecal and intestinal mucosal samples, which are directly connected to the tumor, allowing for a more accurate reflection of the composition of tumor-associated microbiota (48). Thus, to research the tumor-associated microbiota and their functional composition on the mucosal surface, we sequenced 16S rRNA amplicon and metagenomics on the mucosal tissues of CC patients. This study confirmed significant microbial community differences between tumor sites and paired normal mucosa (Fig. 1). In line with other studies, the predominant phyla in the gut microbiota are *Firmicutes*, *Bacteroidetes*, *Fusobacteria*, and *Proteobacteria* (49, 50).

Through metagenomic sequencing with more precise classification and localization, we observed that *Salmonella enterica*, *Klebsiella aerogenes*, *Bacillus cereus*, *Bacillus pseudomycoides*, *Staphylococcus aureus*, *Chlamydia trachomatis*, *Streptococcus pneumoniae*, *Bacillus wiedmannii*, *Mycobacterium tuberculosis*, *Streptococcus dysgalactiae*, *Human endogenous retrovirus*, *Pseudomonas aeruginosa*, *Campylobacter jejuni*, M*ycobacterium* sp. *11000297,* and methanotrophic bacterial endosymbiont of *Bathymodiolus* sp. exhibited relatively high abundance in tumor sites in our cohort. The correlation between *S. enterica* and CC progression has not yet been confirmed. However, studies have reported that infection of *S. enterica* can lead to acute gastroenteritis, and its colonization in the tumor microenvironment can promote CRC progression by secreting intestinal bacterial protein Avra (51). *Klebsiella* is a commensal flora of human and animal gut and is an opportunistic pathogen. An animal experiment showed that the colonization of *K. aerogenes* aggravated colitis by exacerbating the intestinal inflammatory response and destroying the mucosal barrier, which destroyed the intestinal barrier and promoted the tumorigenesis of AOM/DSS mice (52). *B. cereus*, *S. aureus*, and *C. jejuni* are common foodborne pathogens that can lead to food poisoning (53–55). *C. jejuni* has been linked to the occurrence and progression of colon cancer (56). However, the association between *B. cereus*, *S. aureus*, and colon cancer has not been thoroughly studied and confirmed. *M. tuberculosis* is the causative agent of pulmonary tuberculosis (57). Interestingly, Kim et al. discovered a negative relationship between CRC and the incidence of tuberculosis (58). They found that heat-inactivated *M. tuberculosis* could improve inflammation-induced colorectal cancer by altering the composition of intestinal microorganisms. Unlike previous findings, our study reveals that *M. tuberculosis* is enriched in tumor mucosa. Various factors such as sample sources, research methodologies, and experimental conditions could account for this disparity. The abnormal transcriptional activation of *Human endogenous retrovirus* (*HERV*) is associated with the occurrence and progression of cancer (59). Ko et al. found that the expression of *HERV*-K *env* protein was significantly increased in CRC, and knocking out the *HERV*-K *env* gene significantly reduced the tumorigenic characteristics of colorectal cancer cells, including proliferation, invasion, migration, and tumor colonization (60). At present, there is insufficient research evidence to suggest a direct association between *B. pseudomycoides*, *C. trachomatis*, *S. pneumoniae*, *B. wiedmannii*, *S. dysgalactiae*, *P. aeruginosa*, *Mycobacterium* sp. *11000297*, and metamorphotropic bacterial endosymbiont of *Bathymodiolus* sp. with colon cancer. Pathogenic bacteria will be an important target for treating CC, and further research is needed to establish any potential relationship between these tumor-associated microbiota and colon cancer. *Bacteroides vulgatus*, *Bifidobacterium pseudocatenulatum*, *Bifidobacterium breve*, *Bacteroides uniformis*, and *Bacteroides dorei* are enriched in normal mucosa. *B. vulgatus* is one of the predominant species of the *Bacteroides* genus in the human intestinal tract. Research has shown that *B. vulgatus* exhibits tumor-suppressive effects *in vitro*. *B. vulgatus* is one of the predominant species of the Bacteroides genus in the human intestinal tract and is generally considered a beneficial symbiotic bacterium. Research has shown that B. vulgatus exhibits tumor-suppressive effects *in vitro*, and it can alleviate colitis in mice by modulating the gut microbiota and immune response (61, 62). *B. pseudocatenulatum* G7 also exhibits anti-colitis effects (63), but further research is needed to elucidate its association with CC. *B. breve* is also a potential probiotic, and its derived indole-3-lactic acid can improve colitis-associated tumorigenesis by guiding the differentiation of immature colonic macrophages (64). Currently, there is no direct evidence linking *B. uniformis* and *B. dorei* to colon cancer. However, their depletion of tumor mucosa suggests their significant role in intestinal ecological balance. Therefore, further research is needed to substantiate this claim.

To further explore the functional composition of mucosal microbiota, we performed functional annotation of the metagenome. We found that glycoside hydrolases accounted for the majority of differential enzymes, and the abundance of glycoside hydrolase families was significantly decreased in normal mucosa. Significantly, the tumor group exhibited downregulation of genes associated with starch and sucrose metabolism pathways. This suggests the potential role of tumor-associated microbial communities in the pathogenesis of cancer, which may involve starch and sucrose metabolism as well as glycoside hydrolases. Moreover, previous studies also implicated enzymes such as β-galactosidase and β-glucuronidase as key players in the impact of gut microbiota on colorectal cancer (65, 66). In a word, multi-omics analyses can provide a deeper understanding of the role of tumor-associated microbial communities in cancer pathogenesis, and the incorporation of metabolomics and proteomics will be a focus of our future mechanistic studies.

### Identification of the correlated tumor-associated microbiota to DEGs

Consistent with other studies (67, 68), our transcriptomic sequencing results indicate differential gene expression between tumor tissue and adjacent normal tissue. The top three microorganisms most significantly associated with DEGs were metamorphotropic bacterial endosymbiont of *Bathymodiolus* sp., *Bacillus wiedmannii*, and *Campylobacter jejuni* (Fig. S6). However, their role in colon cancer is unknown, and further experimental validation is required to determine whether they impact the host’s transcriptome.

There is a close relationship between metabolic pathways and colon cancer. Abnormalities in metabolic pathways may lead to an imbalance in cell growth, proliferation, and apoptosis, thereby promoting cancer development (69). Increasing evidence suggests that the human gut microbiota exerts carcinogenic effects through its metabolic repertoire, particularly in bile acid metabolism (70). In this study, we observed the enrichment of DEGs that related to differential microbial taxa in bile secretion pathways (Fig. 4). Moreover, we found a strong negative correlation between bacteria belonging to *C. jejuni* and genes associated with bile secretion. Therefore, we hypothesize that *C. jejuni* may promote the development of CC by influencing bile secretion. However, the current research on the relationship between *C. jejuni* and bile secretion is relatively limited. Further in-depth research is required to understand the specific mechanisms and impact of *Campylobacter jejuni* on disease development.

Inflammation mechanisms are key drivers in tumorigenesis, and some CC patients also suffer from inflammatory bowel disease (71). Increased pathogenic or pathobiont microbiota in the gut may lead to excessive activation of the immune system’s inflammatory response, disrupting immune balance and thereby promoting CC development (72). In our study, we analyzed the correlation between immune infiltration and gut microbiota. We found a negative correlation between *C. jejuni* and CD4+ Tem cells, as well as mast cells. Previous studies have indicated that the interaction between the host and the microbiota can influence various aspects of host immunity, including the development, differentiation, and function of immune cells (73, 74). Consistent with this notion, our research has revealed interactions between the microbiota and metabolic pathways. Specifically, we found a correlation between *C. jejuni* and the bile secretion pathway. Therefore, we hypothesize that *C. jejuni* may modulate the host’s immune response by affecting bile metabolism, and their interaction promotes the onset of colon cancer tumors. However, prior studies have suggested that *C. jejuni* can promote the development of colorectal tumors through the action of its cytotoxic distending toxin (56). Our study undoubtedly presents a novel discovery regarding the pro-carcinogenic effect of *C. jejuni*, yet further research is needed to elucidate its underlying mechanism.

### Tumor-associated microbiota—potential therapeutic direction for prolonging CC survival

Microbiota-based therapy is gaining increasing attention in cancer treatment (75, 76). Microbiota-based therapy involves targeted reconstruction of the tumor microbiota or management of microbial metabolites to intervene in cancer, and it is becoming an important component of cancer treatment (6, 47). For example, the addition of physiologically relevant doses of aspirin inhibits the expression of virulence factors FadA/Fap2 in *Fusobacterium nucleatum*, thereby suppressing tumor formation in ApcMin/+ mice induced by *F. nucleatum* (77). Administration of *Bifidobacterium CGMCC 15068* effectively prevents tumor formation in a mouse model of colitis-associated colorectal cancer, and this intervention leads to a marked increase in the abundance of beneficial bacteria in the mouse intestine and induces notable modifications to the metabolic profile of the intestine (78). Our study found that the metabolic bacterial endosymbiont of *Bathymodiolus* sp., *Bacillus wiedmannii*, and *Mycobacterium tuberculosis* are enriched in the tumor mucosa, and they have significant survival value in CC (Fig. 5). However, there is currently no definitive research or evidence to suggest a direct link between these species and CC. Our research findings provide preliminary evidence demonstrating a close association between the metabolic bacterial endosymbiont of *Bathymodiolus* sp., *B. wiedmannii*, *M. tuberculosis*, and survival-related genes in CC. Further exploring this association can help deepen our understanding of their involvement in the development of colon cancer and provide new clues for the treatment and prognosis assessment of CC patients. Future studies could investigate the interactions between these species and survival-related genes in CC, as well as their potential roles in development and treatment response. However, it is important to note that our study is observational and cannot establish a causal relationship; further experimental and clinical research is needed to validate these findings.

However, this study still has some limitations. Firstly, the sample size in this study is limited. In future studies, we will continue to expand the recruitment scope of research subjects, extend the data collection period, and collaborate on multicenter studies to increase the sample size, so that the research results can better describe the microbial landscape of CC. Secondly, the integration of metabolomics and proteomics data into this study could provide a more comprehensive understanding of the molecular mechanisms, revealing the intricate relationships between the gut microbiota and colorectal cancer from multiple levels, identifying potential biomarkers and therapeutic targets, and driving advancements in personalized medicine and translational research. Finally, despite the association between the microbiome and gene expression being discovered, due to the observational nature of the study, the causality of these relationships cannot be determined, necessitating further research for validation. In future studies, we will conduct more in-depth experimental model studies and *in vitro* experiments, integrating multi-omics data to explore the causal mechanisms of microbiome-host interactions.

### Conclusions

Totally, we systematically investigated the special mucosal microbiome and transcriptome profiles in CC. Importantly, the significant correlation between 3,632 DEGs and tumor-associated microbiota in CC was proved. We also found that *Campylobacter jejuni* is related to bile secretion and CD4+ Tem and master cells of immune cells, which may contribute to the occurrence and development of CC. Finally, we also explored the survival value of tumor-associated microbiota on colon cancer, thus providing a new strategy for the treatment of CC. Our integrated analysis of transcriptome and microbiome has revealed potential connections between gut microbiota and host gene expression, contributing to a comprehensive understanding of the interactions between host and microbiota, and providing better strategies for the diagnosis and prevention of CC through further experiments. Moving forward, our research emphasizes the necessity for further studies to unravel the mechanisms by which these microbiota promote cancer. Future research should focus on investigating the complex interactions between these microbiota and host metabolism and immune system, and exploring the potential of harnessing microbiota in cancer treatment.

## Data Availability

A STORMS (Strengthening The Organizing and Reporting of Microbiome Studies) checklist (79) is available at 10.5281/zenodo.14747658. The 16S rRNA, metagenomic, and transcriptome sequencing data have been submitted to the Sequence Read Archive database at the National Center for Biotechnology Information website (https://www.ncbi.nlm.nih.gov/) under accession numbers PRJNA911471, PRJNA1028103 and PRJNA1028158.

## SUPPLEMENTAL MATERIAL

Supplemental Figures msystems.00805-24-s0001.docx Figures S1 to S8.

Supplemental Tables msystems.00805-24-s0002.xlsx Tables S1 to S10.

ASM does not own the copyrights to Supplemental Material that may be linked to, or accessed through, an article. The authors have granted ASM a non-exclusive, world-wide license to publish the Supplemental Material files. Please contact the corresponding author directly for reuse.
