# Supplementary material for: Multi-omics analysis reveals associations between gut microbiota and host transcriptome in colon cancer patients
Source: mSystems. 2025 Feb 27;10(3):e00805-24. doi: 10.1128/msystems.00805-24 (PMC11915798; doi:10.1128/msystems.00805-24)
Supplement: Supplemental Figures — Figures S1 to S8. [file msystems.00805-24-s0001.docx]

Supplementary Material

# Supplementary Figures


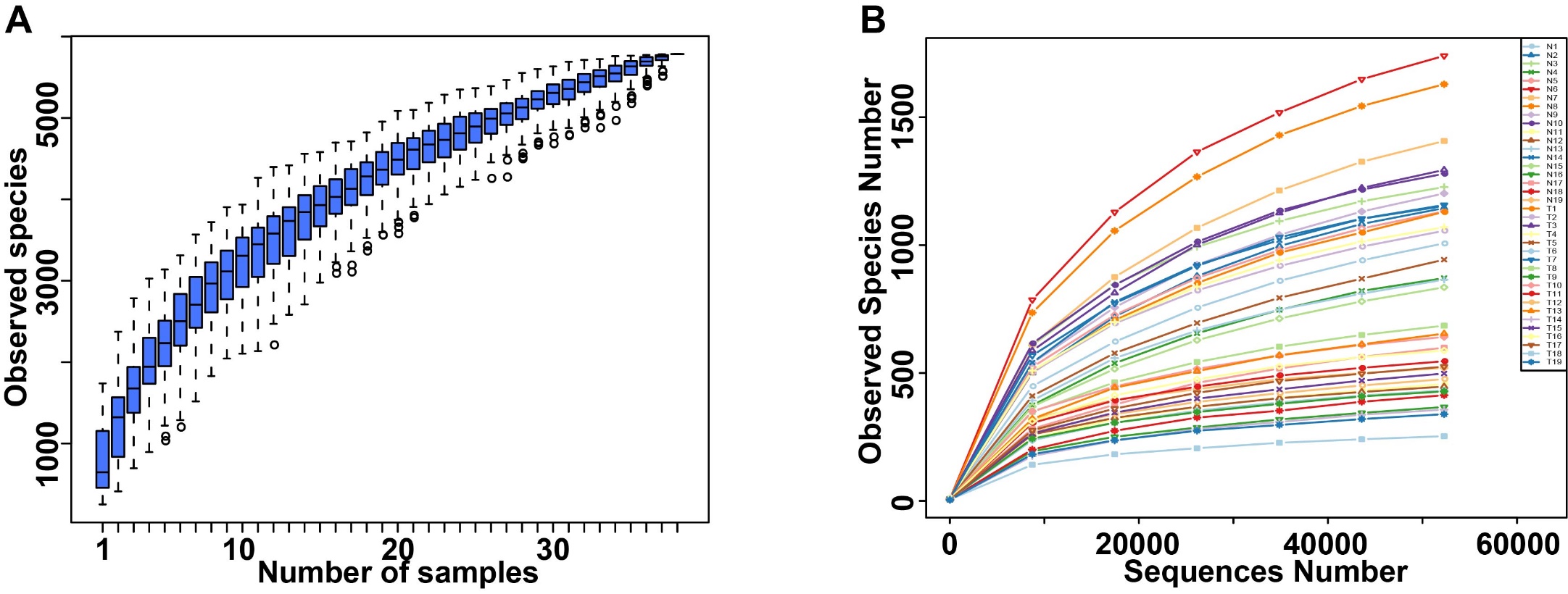


**Supplementary Figure 1 |** The quality control of 16S rRNA sequencing. (A). The species accumulation boxplot for 16S rRNA sequencing. The abscissa is the sample size, and the ordinate is the observed species. (B). The rarefaction curves of 16S rRNA sequencing. The abscissa is the randomly sampled sequencing reads from a specific sample, and the ordinate is the number of OTUs that can be constructed based on those sequencing reads.


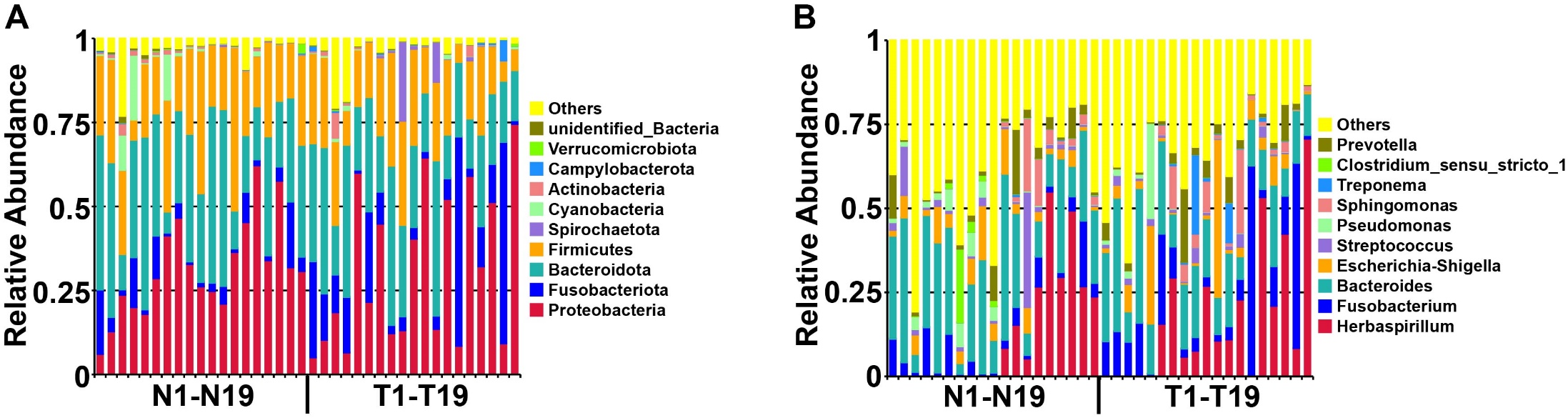


**Supplementary Figure 2 |** The mucosal microbiota composition of all samples. (A) The mucosal microbiota composition of all samples at the phylum level. (B) The mucosal microbiota composition of all samples at the genus level.

**
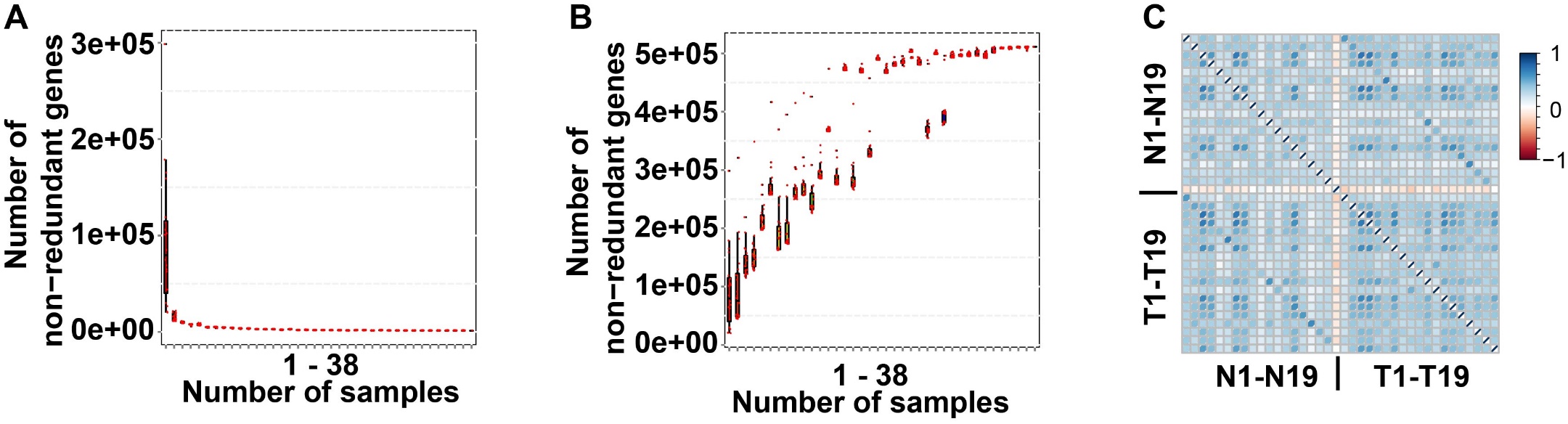
**

**Supplementary Figure 3 |** The quality control of metagenomic sequencing. (A-B) Rarefaction curves of (A) core genes and (B) pan genes. The abscissa is the number of sequencing samples randomly chosen from the sample, and the ordinate is the number of genes. (C) The correlation heatmap of the correlation coefficient between samples. The different colors represent different Spearman correlation coefficients.


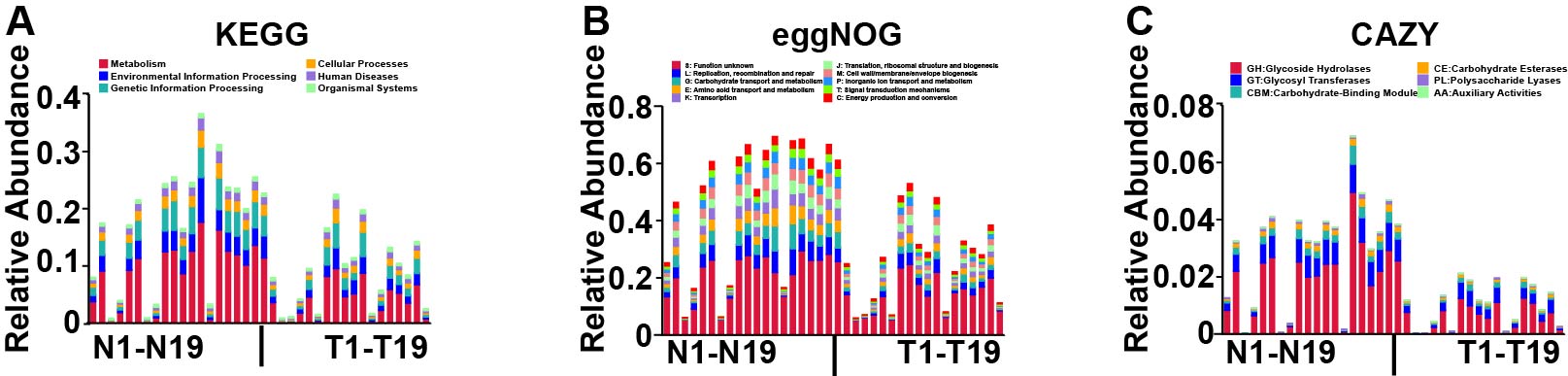


**Supplementary Figure 4 |** At the first classification level, the functions composition of mucosal microbiota in all samples based on KEGG (A), eggNOG (B), and CAZy (C) databases.

**
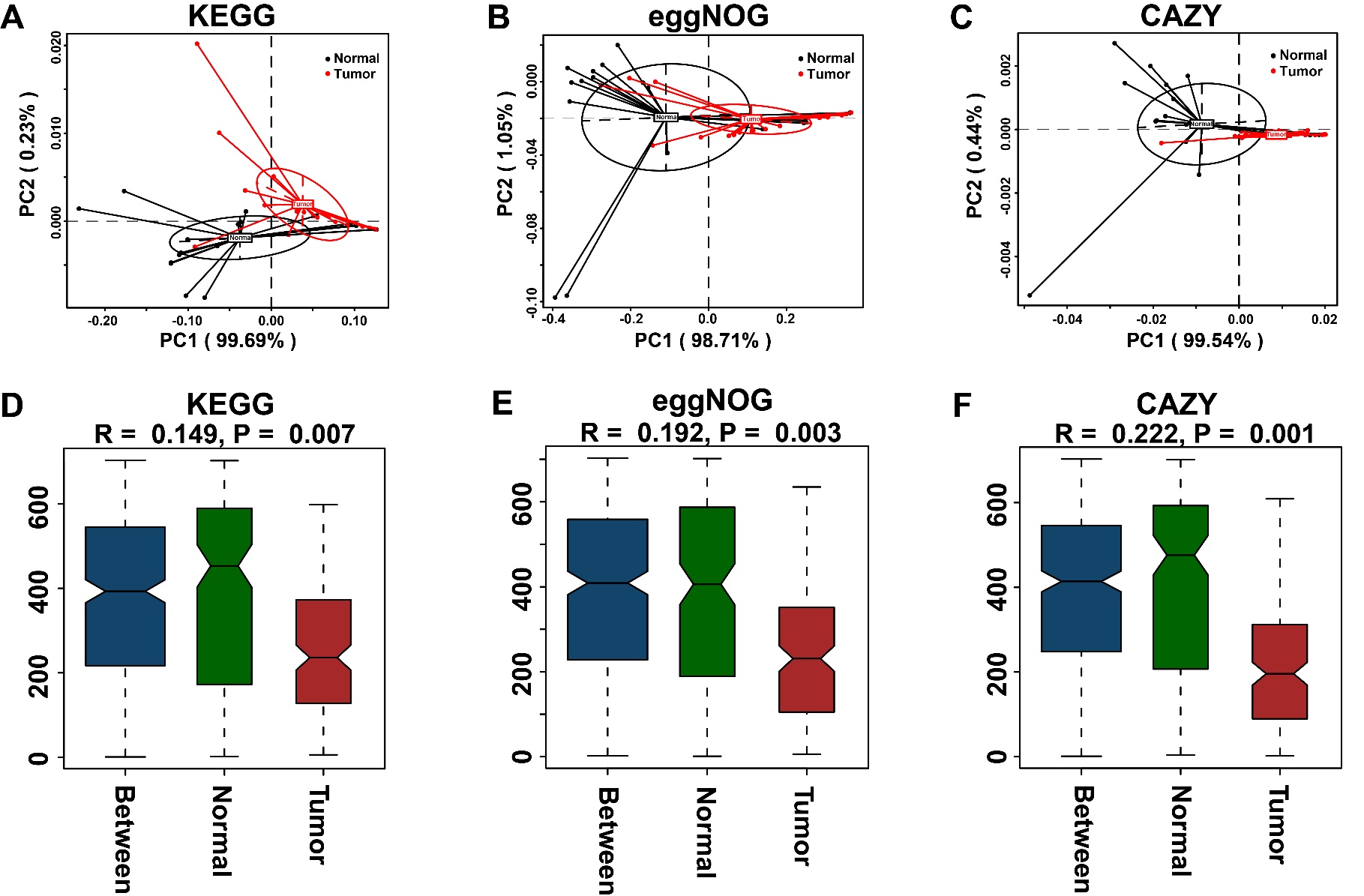
Supplementary Figure 5** | At the first classification level, principal coordinate analysis (PCoA) results are based on KEGG (A), eggNOG (B), and CAZy (C) databases. At the first classification level, Analysis of Similarities (ANOSIM) results are based on KEGG (D), eggNOG (E), and CAZy (F) databases.

**
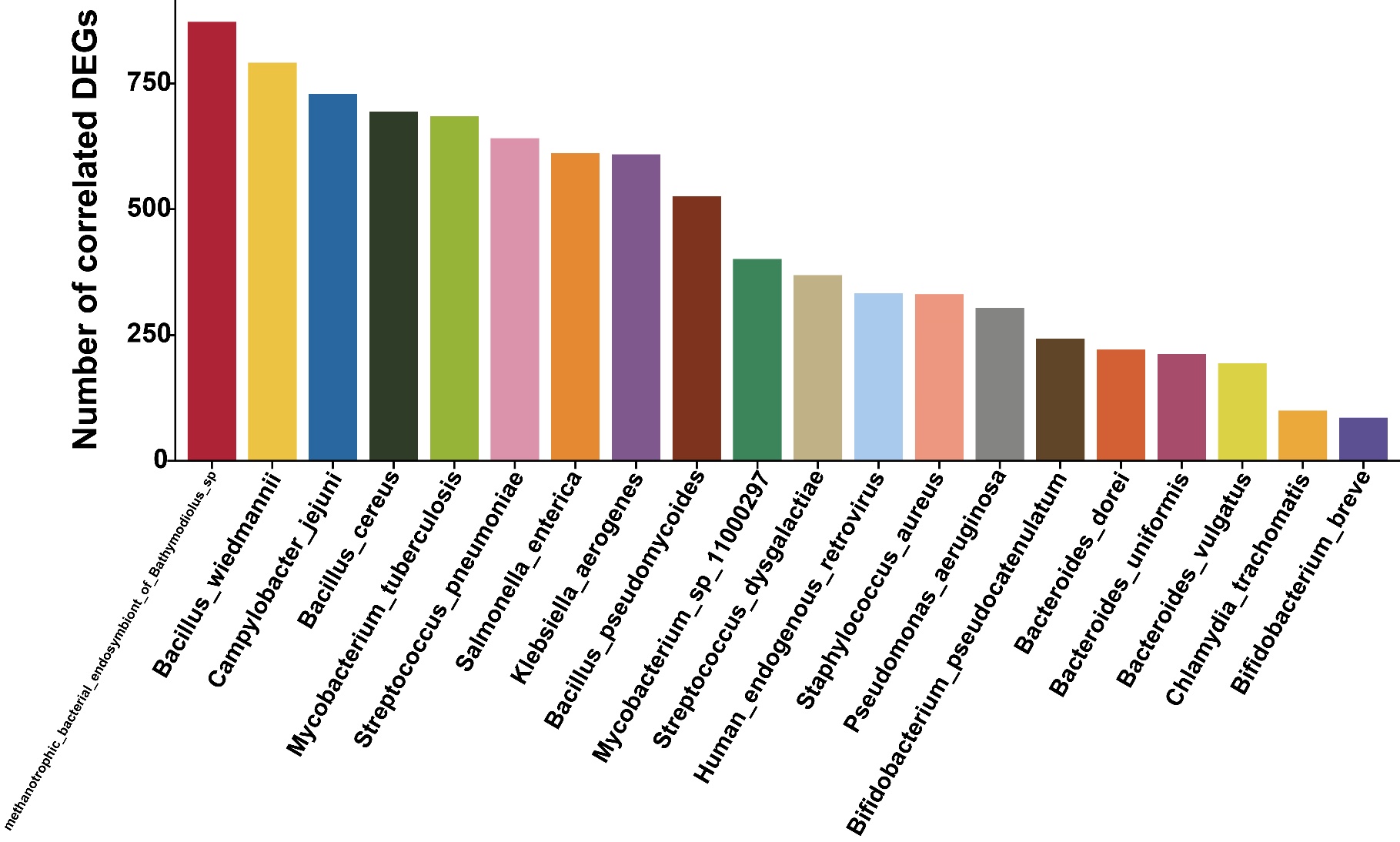
**

**Supplementary Figure 6** | The number of DGEs correlated to tumor-associated microbiota.


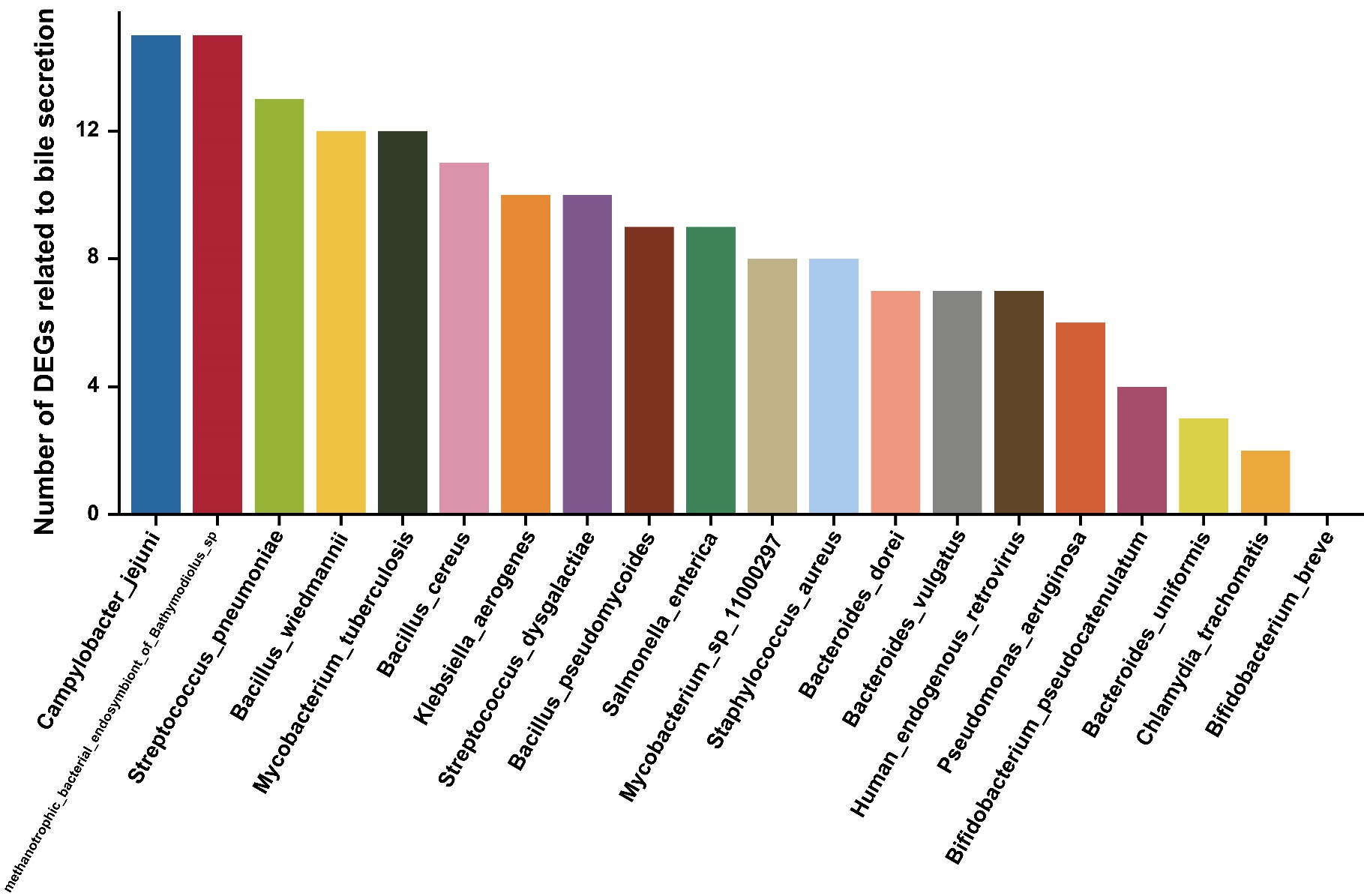


**Supplementary Figure 7** | The number of DGEs related to bile secretion correlates with tumor-associated microbiota.

**
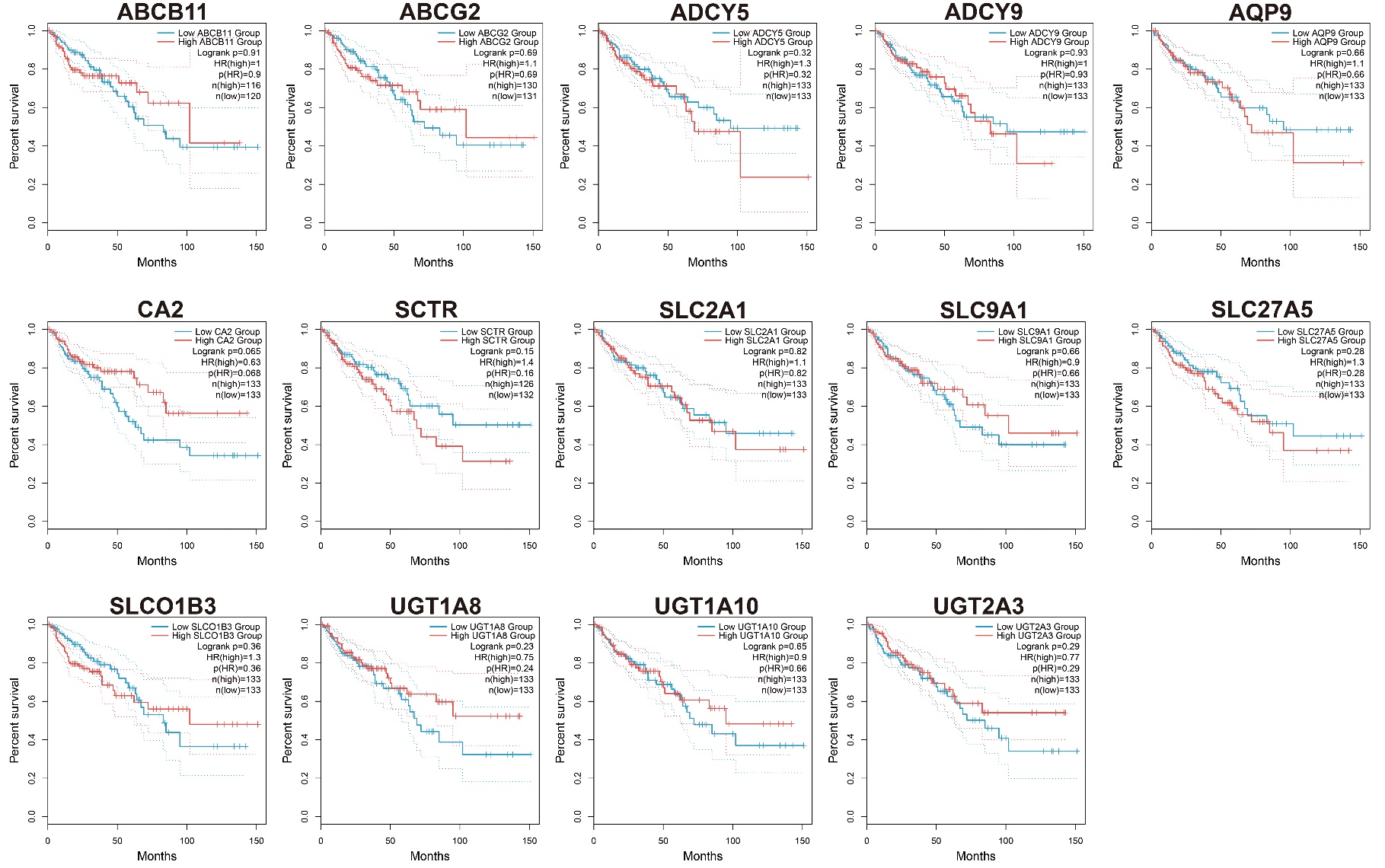
**

**Supplementary Figure 8** | Survival analysis of bile secretion-related DEGs in CC. The result of *SLC10A2* and *UGT2B11* was for absence, due to insufficient sample size at these custom thresholds.
